# The complete mitochondrial genome of *Callionymus olidus* (Perciformes Callionymidae)

**DOI:** 10.1080/23802359.2020.1745098

**Published:** 2021-01-15

**Authors:** Yanshan Liu, Yanping Wang, Jiawen Yin, Daming Li, Shengkai Tang, Xiankun Gu, Tongqing Zhang, Haoran He, Binqing Zhu

**Affiliations:** a Key Laboratory of Fisheries Resources in Inland Water of Jiangsu Province, Freshwater Fisheries Research Institute of Jiangsu Province, Nanjing, People’s Republic of China; b Marine Fisheries Research Institute of Jiangsu Province, Nantong, People’s Republic of China; c Research Center for Nature Conservation and Biodiversity of Nanjing Institute of Environmental Sciences, Ministry of Ecology and Environment, Nanjing, People’s Republic of China

**Keywords:** Mitochondrial genome, *Callionymus olidus*, Callionymidae, phylogenetic tree

## Abstract

In this study, we present the complete mitogenome and a phylogenetic analysis of *Callionymus olidus*, determined by long PCR and primer walking methods. The complete mitochondrial genome is a circular molecule of 16,491 bp in length and contains the same set of 37 mitochondrial genes (13 protein-coding genes, 2 ribosomal RNA (rRNA), 22 transfer RNA (tRNA)), and a control region as other bony fishes. The base composition of the entire mitogenome showed a slight excess of AT bias. The entire mitogenome data produced in this study provides the genomic resources available for future evolutionary studies.

*Callionymus olidus* belongs to the order Perciformes, and is a common small with low-value species in China. It is mainly distributed in the East China Sea, coast waters of the South China Sea, and the lower reaches of rivers, Korean Peninsula (Ni and Wu 2006). It is a small fish living in warm bottom water and inhabit in the seawater or pure fresh water areas in the lower reaches of rivers. No research on *C. olidus* has been documented till date. Therefore, it is highly important to obtain the complete mitochondrial genome of *C. olidus* for further studies.

In the present study, one *C. olidus* specimen (NO. LX_JJ190624) was collected from the Jingjiang Reach of Yangtze River (120°28′52.35″E, 32°3′40.26″N) on 24 June 2019 and stored in the herbarium of Freshwater Fisheries Research Institute of Jiangsu Province.

The complete mitogenome of *C. olidus* (NO. LX_JJ190624) is sequenced to be 16,491 bp in length (GenBank accession number: MN830944 submitted for review) and shares high similarity with the gene content and structure of most vertebrates (Wolstenholme 1992; Boore 1999). It consists of 13 typical vertebrate protein-coding genes, 22 tRNA genes, 2 rRNA genes (12S rRNA and 16S rRNA), and 2 major non-coding regions (control region and L-strand replication origin). Most mitochondrial genes of *C. olidus* are encoded on the H-strand, except for ND6 and eight tRNA (Gln, Ala, Asn, Cys, Tyr, Ser-UCN, Glu, and Pro) genes that are encoded on the L-strand. The overall nucleotide composition of the heavy strand is 29.09% T, 26.03% C, 28.52% A, and 16.35% G, with anti-G bias and a slight excess of AT as the observation in most fishes (Miya et al., 2001, 2003). The 13 protein-coding genes are totally 11,388 bp in size, accounting for 69.06% of the whole mitogenome. The *C. olidus* mitogenome also contains a small subunit rRNA (12S rRNA) and a large subunit rRNA (16S rRNA), which are 942 and 1657 bp in length, respectively.

To investigate the phylogenetic relationships of *C. olidus* and other 12 species from Perciformes, phylogenetic trees is obtained using maximum-likelihood (ML) analyses based on entire COI sequences. Two species in the order Scorpaeniformes are used as outgroup (Figure 1). The topology of phylogeny trees for 13 Perciformes species shows that suborder Callionymoidei is a single group. Unexpectedly, the two species in suborder Percoidei are separated. As a few sequences of the suborder Calionymoidei can be downloaded from the GenBank, more details of the topology of phylogeny trees need to be study and further investigation ise needed to reveal the reason for this unexpected phenomenon.

**Figure 1.**
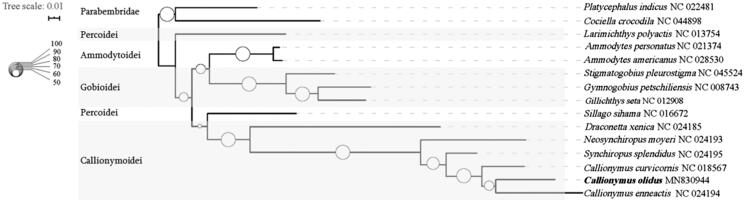
Phylogenetic tree using the COI sequences. The sequences are downloaded from GenBank and the phylogenic tree is constructed by maximum-likelihood (ML) method. The bootstrap values and posteriori probability were presented as the proportion of white circles, respectively. The genomic sequences obtained in this study are shown by bold type.
